# Decidual Interleukin-22-Producing CD4+ T Cells (Th17/Th0/IL-22+ and Th17/Th2/IL-22+, Th2/IL-22+, Th0/IL-22+), Which Also Produce IL-4, Are Involved in the Success of Pregnancy

**DOI:** 10.3390/ijms20020428

**Published:** 2019-01-19

**Authors:** Federica Logiodice, Letizia Lombardelli, Ornela Kullolli, Herman Haller, Enrico Maggi, Daniel Rukavina, Marie-Pierre Piccinni

**Affiliations:** 1 Department of Experimental and Clinical Medicine and DENOTHE Excellence Center, University of Florence, 50134 Florence, Italy; federica_logiodice@yahoo.it (F.L.); letizialombardelli@gmail.com (L.L.); ornelahysa@hotmail.it (O.K.); enrico.maggi@unifi.it (E.M.); 2 Department of Gynecology and Obstetrics, Medical Faculty, University of Rijeka, 51000 Rijeka, Croatia; herman.haller@medri.uniri.hr; 3 Immunology Area, IRCCS Bambino Gesù Children’s Hospital, 00165 Rome, Italy; 4 Department of Physiology and Immunology, Medical Faculty, University of Rijeka, 51000 Rijeka, Croatia; rimed@hazu.hr

**Keywords:** Th22, Th17, pregnancy, IL-22, IL-4, IL-5, IL-17, spontaneous abortion, ectopic pregnancy, implantation

## Abstract

Trophoblast expressing paternal HLA-C resembles a semiallograft, and could be rejected by maternal T cells. IL-22 seems to be involved in allograft rejection and thus could be responsible for miscarriages. We examined the role of decidual IL-22-producing CD4+ T on human pregnancy. In those experiencing successful pregnancy and those experiencing unexplained recurrent abortion (URA), the levels of IL-22 produced by decidual CD4+ T cells are higher than those of peripheral blood T cells. We found a correlation of IL-22 and IL-4 produced by decidual CD4+ T cells in those experiencing successful pregnancy, not in those experiencing URA. The correlation of IL-22 and IL-4 was also found in the serum of successful pregnancy. A prevalence of CD4+ T cells producing IL-22 and IL-4 (Th17/Th2/IL-22+, Th17/Th0/IL-22+, Th17/Th2/IL-22+, and Th0/IL-22+ cells) was observed in decidua of those experiencing successful pregnancy, whereas Th17/Th1/IL-22+ cells, which do not produce IL-4, are prevalent in those experiencing URA. Th17/Th2/IL-22+ and Th17/Th0/IL-22+ cells are exclusively present at the embryo implantation site where IL-4, GATA-3, IL-17A, ROR-C, IL-22, and AHR mRNA are expressed. T-bet and IFN-γ mRNA are found away from the implantation site. There is no pathogenic role of IL-22 when IL-4 is also produced by decidual CD4+ cells. Th17/Th2/IL-22+ and Th17/Th0/IL-22+ cells seem to be crucial for embryo implantation.

## 1. Introduction

The conceptus, because of the presence of paternal classical MHC class I antigens (HLA-C) [1], is thought to resemble a semiallograft [2]. The maternal effector CD4+ T helper lymphocytes activated by the paternal antigens expressed by trophoblast, after presentation by maternal APCs, can secrete various cytokines. 

On the basis of the cytokines produced, the human effector CD4+ T helper cells have been classified as T helper (Th)1 and Th2, and, more recently, as Th17 cells [3,4] and Th22 cells [5]. Indeed, CD4+ Th1 cells, which produce interleukin (IL)-2 and interferon (IFN)-γ, are highly protective against infections sustained by intracellular pathogens. CD4+ Th2 cells, which are mainly responsible for host defense against extracellular parasites, including nematodes, produce IL-5, IL-13, and IL-4 [3]. Th17 cells produce IL-17A, IL17F, IL-21, IL-26, and IL-22 [5,6,7] and express retinoic-acid-related orphan receptor (ROR)-C [8]. They are highly protective against infections by extracellular bacteria by recruiting neutrophils, which contribute to the chronic tissue inflammation. Precursors of Th17 cells can differentiate into Th17/Th1, producing both IL-17 and IFN-γ, and finally into Th1 cells, in response to IL-12 present in the microenvironment [9], whereas an IL-4-rich microenvironment may induce the shifting of memory Th17 cells into Th17/Th2 cells, producing both IL-17 and IL-4 [10].

Th22 cells primarily secrete IL-22 and TNF-alpha and could express granzyme B and IL-13, factors associated with host defense and remodeling of tissue [11]. The expansion of IL-22-producing cells appears to be regulated by the aryl hydrocarbon receptor (AHR) transcription factor, although additional intracellular molecules involved in Th22 differentiation as STAT3, are also required by Th17 cells [12]. Expression of the CCR4 and CCR10 skin-homing receptors on Th22 cells suggests these cells are likely recruited to the skin. Indeed Th22 cells may be involved in the pathogenesis of inflammatory skin disorders such as psoriasis, atopic eczema, and allergic contact dermatitis [5,13,14]. In vitro Th22 cells may develop independently of the Th17 lineage while demonstrating plasticity toward Th1- and Th2-type cells [11]. Under Th1-promoting conditions in vitro, as well as in an IFN-γ–rich inflammatory environment in vivo, Th22 cells display marked plasticity toward the production of IFN-γ, further supporting an important role for T-bet in Th22 cell function. Th22 cells also exhibit plasticity under Th2 culture conditions in vitro, with increased IL-13 expression. Consistent with these results, skin-homing IL-13- and IL-22-producing Th2/IL-22 and Tc2/IL-22 cells were recently found to be elevated in people with atopic dermatitis (AD) [15,16,17]. 

Importantly for pregnancy, the Th1 cells (in particular IFN-γ) seem to play a role in acute fetal allograft rejection and thus could be involved in recurrent spontaneous abortion (URA) [18,19,20,21,22,23], whereas Th2 cells [20] (producing IL-4 and IL-10) and CD4+CD25+Foxp3+ T reg cells (producing IL-10 and TGF beta), inhibiting IFN-γ production, act to enhance fetal allograft tolerance [24] and thus could be responsible for the success of pregnancy. Very recently, we observed a prevalence of Th17/Th2 cells (producing IL-17 and IL-4) in the decidua of those experiencing successful pregnancy, whereas the presence of Th17 (producing IL-17 only) and Th17/Th1 (producing IL-17 and IFN-γ) cells was exclusively found in the decidua of those experiencing unexplained recurrent abortion [25]. More interestingly, Th17/Th2 cells were exclusively present at the embryo implantation site, and IL-4, GATA-3, IL-17A, and ROR-C mRNA levels increased at embryo implantation sites, whereas Th17, Th17/Th1, and Th1 cells were exclusively present apart from implantation sites [25]. Moreover, we found that HLA-G5, a soluble Class I b molecule released by embryo and extravillous trophoblast, could be responsible for the development of Th17/Th2 cells [25].

IL-22 has been shown to be involved in allograft rejection, by increasing IFN-γ production by Th1 and Tc1 cells, known to be responsible for acute allograft rejection, and decreasing IL-10 production by T reg and Th2 cells, known to be responsible for allograft tolerance [26,27]. Because of this, IL-22 produced by CD4+ T cells from decidua could be a factor responsible for miscarriage. Only two discordant publications have described the possible role of IL-22 on URA: one indicates that IL-22 could be responsible for URA because serum IL-22 levels increase compared to cases of successful pregnancy, and the other reports that mRNA for IL-22 decreases in decidua of those experiencing URA compared to successful pregnancy [28,29].

In human early pregnancy decidua, the ILC3 were shown to release IL-22 [30] and could partially differentiate into uterine NK cells. Until now, the role of IL-22-producing CD4+ T cells on human pregnancy has never been documented and has to be clarified. 

We generated CD4+ T clones from peripheral blood and decidua of those experiencing successful pregnancy (elective termination pregnancy in the first trimester), URA at the moment of a spontaneous abortion during the first trimester of pregnancy, and ectopic pregnancy (at both embryo implantation site in the Fallopian tube and distant from that site). By measuring IL-22 but also Th1-type, Th2-type, and Th17-type cytokines, which could be associated with the production of IL-22, we analyzed the different CD4+ Th22-type profiles and the different Th22-like subpopulations at the fetomaternal interface and at the embryo implantation site. Thus, in this study we (i) defined the role of Th cells producing IL-22 in pregnancy, (ii) defined to which Th subpopulation (of Th1-, Th2-, Th0-, Th17-, Th17/Th1-, Th17/Th2, and Th17/Th0 cells) the decidual Th cells producing IL-22 belong, ascertaining the particular profiles preferentially associated with pregnancy failure or successful pregnancy, and (iii) found the particular Th22-type profiles which could preferentially favor embryo implantation.

## 2. Results 

### 2.1. Associated Production of IL-4, IL-13, and IL-22 by Decidual CD4+ T Cell Clones in Those Experiencing Successful Pregnancy

One hundred twenty-two CD4+ T cell clones were respectively generated from decidual biopsies, and peripheral blood was obtained from nine pregnant women (with successful pregnancy) who voluntarily underwent an elective termination of pregnancy; 125 CD4+ T cell clones were respectively generated from decidual biopsies, and peripheral blood was obtained from four women suffering from unexplained recurrent abortion (Experiment 1 in Section 4.3). IL-4, IL-13, IL-5, IL-17A, IL-17F, IL-22, and IFN-γ were measured in the supernatant of the CD4+ T cell clones by a multiplex bead-based assay.

**Figure 1:**
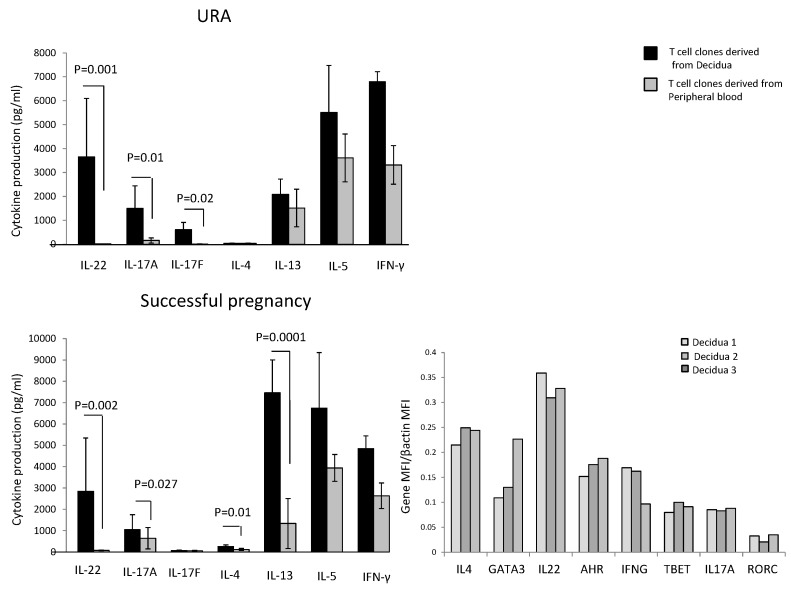
Cytokine production by CD4+ T cell clones derived from decidua of those experiencing successful pregnancy and URA and mRNA expression of cytokines and transcription factors in decidual biopsies of successful pregnancy. CD4+ T cell clones were generated from decidual biopsies, and peripheral blood was obtained from those experiencing successful pregnancy and those experiencing unexplained recurrent abortion (URA) (Experiment 1 in Section 4.3). IL-4, IL-13, IL-5, IL-17A, IL-17F, IL-22, and IFN-γ were measured in the supernatant of the CD4+ T cell clones by a multiplex bead-based assay. The statistical analysis was performed with the Wilcoxon test. The determination of mRNA level for IL-4, GATA-3, IL-17A, ROR-C, IL-22, AHR, T-bet, and IFN-γ in three biopsies of decidua from three pregnant women (with successful pregnancy) was performed by Quantigene 2.0.

In those experiencing successful pregnancy, decidua CD4+ T cell clones produce higher levels of IL-4 (*p* = 0.01), IL-13 (*p* = 0.0001) (two Th2-type cytokines), IL-22 (*p* = 0.002) (a Th17/Th22-type cytokine), and IL-17A (*p* = 0.027) (one of the two Th17-type cytokines), but not higher levels of IL-17F and IL-5 compared to peripheral blood T cell clones (Figure 1). By contrast, IFN-γ production by T cell clones was not statistically different in the decidua compared to peripheral blood (Figure 1). 

In those experiencing URA, decidua CD4+ T cell clones do not produce IL-4, but produce higher levels of IL-22 (*p* = 0.001), IL-17A (*p* = 0.01), and IL-17F (*p* = 0.02) compared to peripheral blood T cell clones (Figure 1). By contrast, IFN-γ, IL-5, and IL-13 production by T cell clones was not statistically different in the decidua compared to peripheral blood (Figure 1). 

These results show that there is an accumulation of CD4+ T cells producing IL-17A, IL-17F, and IL-22 in the decidua of those experiencing URA and an accumulation of T helper cells producing IL-17A, IL-22, IL-13, and IL-4 in the decidua of those experiencing successful pregnancy, suggesting an associated production of IL-4, IL-13, and IL-22 by decidual CD4+ T cells in those experiencing successful pregnancy, not found in those experiencing URA.

We also measured the mRNA expression of IL-4 and its associated transcription factor GATA3, IL-17A and its associated transcription factor RORC, and IL-22 and its associated transcription factor AHR directly on decidual biopsies of successful pregnancy. IL-17A, IL-22, IL-4, and their associated transcription factors RORC, AHR, and GATA3 mRNAs are expressed in the decidua of those experiencing successful pregnancy (Figure 1). We confirm the association of IL-22 and IL-4 at the mRNA level in the decidua of those experiencing successful pregnancy.

### 2.2. In Those Experiencing Successful Pregnancy, IL-22 Is Positively Correlated with the Th2-Type Cytokine IL-4, Whereas, in those Experiencing URA, IL-22 Produced by CD4+ T Cell Clones Derived from the Decidua Is Positively Correlated with Th17-Type Cytokines (IL-17A and IL-17F) 

The levels of IL-22 and the levels of IL-4, IL-13, IL-5, IL-17A, IL-17F, and IFN-γ measured in the supernatants of the CD4+ T cell clones derived from deciduae of those experiencing URA and those experiencing successful pregnancy have been correlated.

**Figure 2:**
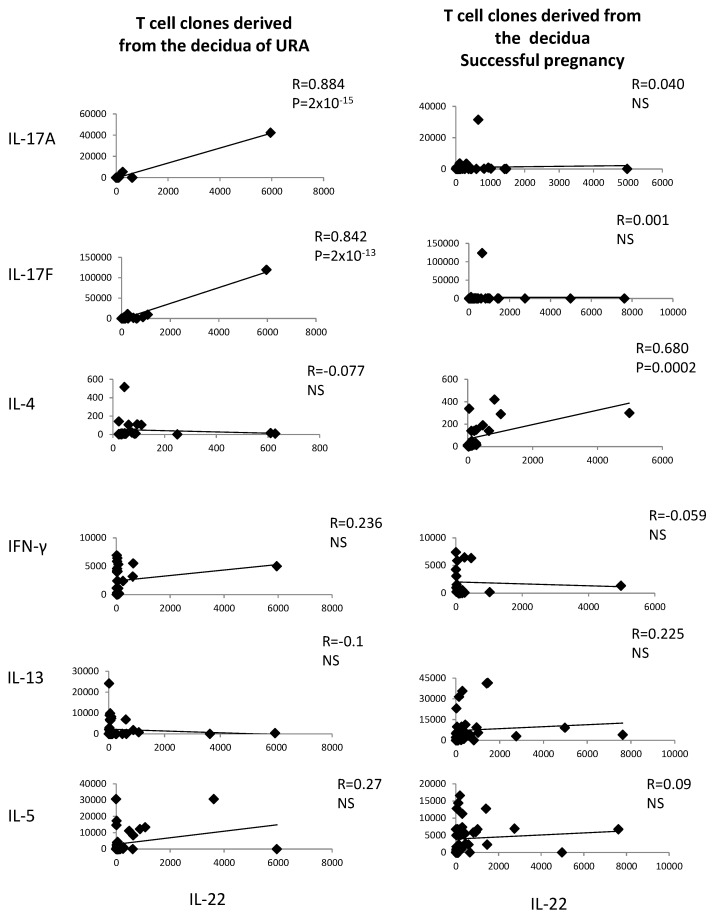
In those experiencing successful pregnancy, IL-22 is positively correlated with IL-4, whereas, in those experiencing URA, IL-22 produced by CD4+ T cell clones derived from the decidua is positively correlated with IL-17A and IL-17F. The levels of IL-22 and the levels of IL-4, IL-13, IL-5, IL-17A, IL-17F, and IFN-γ measured in the supernatants of the CD4+ T cell clones derived from deciduae of those experiencing unexplained recurrent abortion (URA) and those experiencing successful pregnancy (Experiment 1 in Section 4.3) have been correlated. NS means that p is not significant.

IL-22 produced by decidual CD4+ T cells of those experiencing successful pregnancy is positively correlated with IL-4 produced by the same cells (*R* = 0.680, *p* = 0.0002) (Figure 2), whereas, in those experiencing URA, IL-22 is positively correlated with IL-17A and IL-17F, but not with IFN-γ, IL-13, or IL-4 (Figure 2). IL-22 is not correlated with IL-13, IL-5, IL-17A, IL-17F, or IFN-γ in those experiencing successful pregnancy (Figure 2). 

These results confirm the associated production of IL-22 and IL-4, but not the associated production of IL-22 and other Th2-type cytokines (IL-13 and IL-5), by decidual CD4+ T cells of successful pregnancy suggested in Figure 1 and show an associated production of IL-22 and Th17-type cytokines by decidual CD4+ T cells in those experiencing URA.

### 2.3. In Serum of Successful Pregnancy, IL-22 Is Positively Correlated with IL-4 

**Figure 3:**
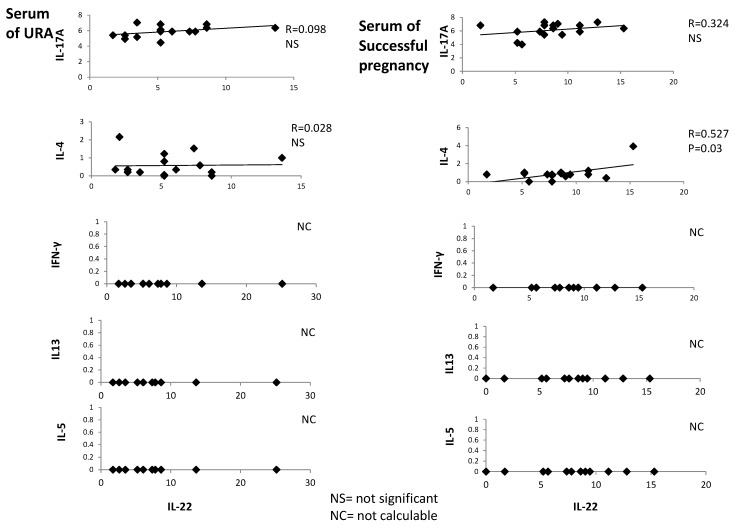
In the serum of successful pregnancy, IL-22 is positively correlated with IL-4. The levels of IL-22, IL-4, IFN-γ, IL-5, IL-13, and IL-17A were measured with a multiplex bead-based assay in the serum of 18 women with successful pregnancy and 18 unexplained recurrent abortion (URA) patients, and these levels were correlated.

IL-22, IL-4, IFN-γ, IL-5, IL-13, and IL-17A were measured with a multiplex bead-based assay in the serum of 18 women with successful pregnancy and 18 URA patients. IL-22 is positively correlated with IL-4 in the serum of those experiencing successful pregnancy (*R* = 0.527, *p* = 0.03) (Figure 3), whereas serum IL-22 is not correlated with serum IL-13, IL-5, IL-17A, and IFN-γ in those experiencing successful pregnancy. Serum IL-22 is not correlated with serum IL-4, IL-13, IL-5, IL-17A, and IFN-γ in those experiencing URA (Figure 3).

These data confirm the associated production of IL-22 and IL-4 in those experiencing successful pregnancy, as suggested by Figure 1 and Figure 2.

### 2.4. The Prevalence of CD4+ T Helper Cells Producing IL-22 (Th/IL-22+) in the Decidua of Those Experiencing Successful Pregnancy

The role of IL-22 on pregnancy is not clear. It has been reported that IL-22 could be associated with URA because, in the serum of URA, IL-22 levels increase compared to cases of successful pregnancy [29]. However, other authors reported a prevalence of IL-22 in those experiencing successful pregnancy because mRNA for IL-22 decreases in decidua of those experiencing URA compared to those experiencing successful pregnancy [28]. To determine if IL-22 production by decidual CD4+ T cells is associated with successful pregnancy or URA, we derived 92 CD4+ T cell clones respectively from decidua of those experiencing successful pregnancy and URA (as in Experiment 1 in Section 4.3). IL-22 was measured in the supernatant of the CD4+ T cell clones by a multiplex bead-based assay.

**Figure 4:**
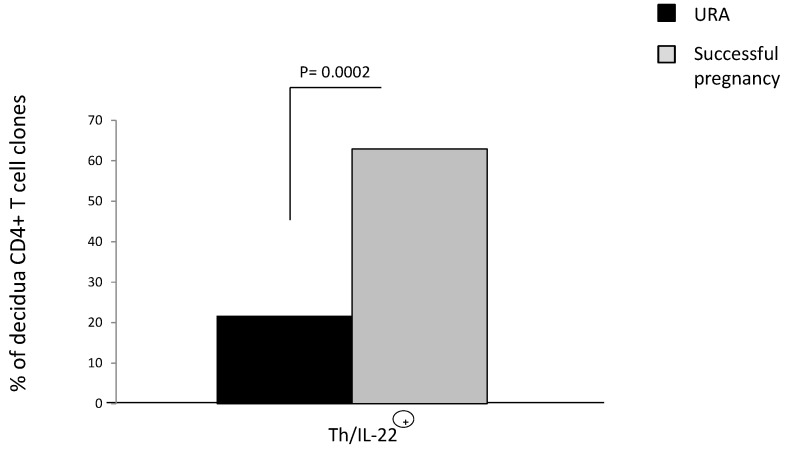
Prevalence of CD4+ T helper cells producing IL-22 (Th/IL-22+) in the decidua of those experiencing successful pregnancy. The levels of IL-22 and the levels of IL-4, IL-13, IL-5, IL-17A, IL-17F, and IFN-γ have been measured in the supernatants of the CD4+ T cell clones derived from deciduae of those experiencing unexplained recurrent abortion (URA) and those experiencing successful pregnancy (Experiment 1 in Section 4.3). The percentage of CD4+ T cells producing IL-22 (Th/IL-22+) in the decidua of those experiencing successful pregnancy and the percentage of T helper cells producing IL-22 in the URA deciduae have been calculated. The statistical analysis was performed with chi-square test.

The percentage of CD4+ T cells producing IL-22 (Th/IL-22+) in the decidua of those experiencing successful pregnancy (62.9%) is higher than the percentage of T helper cells producing IL-22 in the URA deciduae (21.6%) (*p* = 0.002) (Figure 4), suggesting that there is a prevalence of CD4+ T helper producing IL-22 in the decidua of those experiencing successful pregnancy and that IL-22 could have a positive effect on pregnancy.

### 2.5. T Helper Subpopulations Producing IL-22 Preferentially Associated with Pregnancy Failure and Successful Pregnancy

**Figure 5:**
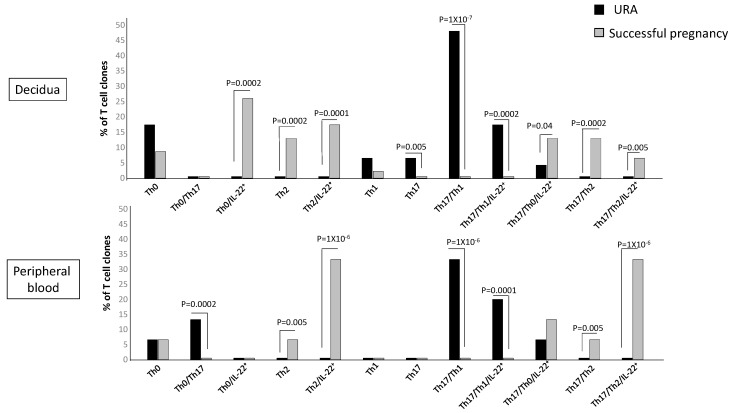
T helper subpopulations present in the decidua and in the peripheral blood of those experiencing unexplained recurrent abortion and those experiencing successful pregnancy. To investigate the CD4+ cell subsets that produce IL-22, the percentages of Th1-, Th2-, Th0-, Th17-, Th17/Th1-, Th17/Th2-, and Th17/Th0 cells, which do not produce IL-22 and which also produce IL-22 (Th1/IL-22+, Th2/IL-22+, Th0/IL-22+, Th17/IL-22+, Th17/Th1/IL-22+, Th17/Th2/IL-22+, and Th17/Th0/IL-22+) were analyzed. Cytokines were measured in the supernatants of the CD4+ T cell clones derived from the decidua and peripheral blood of those experiencing successful pregnancy and those experiencing unexplained recurrent abortion (URA) (Experiment 1 in Section 4.3) by a multiplex bead-based assay. The statistical analysis was performed with the chi-square test.

The previous figure did not show to which CD4+ T helper subpopulation(s) the decidual Th/IL-22+-cells belong and if particular T helper profiles are preferentially associated with pregnancy failure or successful pregnancy. To investigate the CD4+ cell subsets that produce IL-22, we analyzed the percentages of Th1-, Th2-, Th0-, Th17-, Th17/Th1-, Th17/Th2-, and Th17/Th0 cells, which also produce IL-22 (Th1/IL-22+, Th2/IL-22+, Th0/IL-22+, Th17/IL-22+, Th17/Th1/IL-22+, Th17/Th2/IL-22+, and Th17/Th0/IL-22+). We analyzed these subpopulations not only at decidual level but also in the peripheral blood to investigate if some or all of these IL-22-producing T cell subpopulations could be present in peripheral blood or only at the fetomaternal interface. One hundred twenty-five CD4+ T cell clones were derived respectively from the decidua and peripheral blood of those experiencing successful pregnancy and those experiencing unexplained recurrent abortion (Experiment 1 in Section 4.3) (Figure 5).

There are some CD4+ Th subpopulations that do not produce IL-22 but are prevalent in the decidua of those experiencing URA, the Th17 cells, and Th17/Th1, confirming our previous report [25]. The Th17/Th1 cells, but not the Th1 cells, are also prevalent in the URA peripheral blood. There are also some Th subpopulations that do not produce IL-22 but are prevalent in the decidua and in the peripheral blood of those experiencing successful pregnancy, the Th2 cells, and Th17/Th2, confirming our previous reports [25,31]. 

The CD4+ subpopulations that produce IL-22 in the decidua and in the peripheral blood of those experiencing successful pregnancy or URA are Th0, Th2, Th17/Th1, Th17/Th0, and Th17/Th2 cells.

The percentage of Th0/IL-22+ (producing IFN-γ, IL-4, IL-5, IL-13, and IL-22 (84%), producing IFN-γ, IL-4, IL-5, and IL-22 (8%), and producing IFN-γ, IL-4, IL-13, and IL-22 (8%)) (*p* = 0.0002) (Table 1), of Th2/IL-22+ (all producing IL-4, IL-5, IL-13, and IL-22) (*p* = 0.0001) (Table 1), of Th17/Th0/IL-22+ (producing IFN-γ, IL-4, IL-5, IL-13, and IL-22 (66%), producing IFN-γ, IL-4, IL-5, and IL-22 (17%), and producing IFN-γ, IL-4, IL-13, and IL-22 (17%)) (*p* = 0.04) (Table 1), and of Th17/Th2/IL-22+ (all producing IL-4, IL-17A, IL-17F, IL-5, IL-13, and IL-22) (*p* = 0.005) (Table 1) clones are significantly higher in the decidua of those experiencing successful pregnancy compared to the decidua of women suffering from URA (Figure 5). In contrast, the percentage of Th17/Th1/IL-22+ clones+ (producing IFN-γ, IL-17A, IL-17F, and IL-22, but not IL-4) (*p* = 0.005) (Table 1) is significantly higher in the decidua of those experiencing URA compared to those experiencing successful pregnancy (Figure 5). 

In peripheral blood, contrarily to decidua, the percentage of Th0/IL-22+ cells (producing IFN-γ, IL-4, IL-5, IL-13, and IL-22) and the percentage of Th17/Th0/IL-22+ cells (producing IFN-γ, IL-4, IL-5, IL-13, IL-17A, IL-17F, and IL-22) are not statistically different in those experiencing URA and those experiencing successful pregnancy (Figure 5).

However, as for decidua, the percentage of Th2/IL-22+ (producing IL-4, IL-5, and IL-22 with IL-13 (20%), without IL-13 (80%)) (*p* = 0.000001) and the percentage of Th17/Th2/IL-22+ (producing IL-4, IL-17A, IL-17F, and IL-22 (20%), with IL-5 and IL-13 (20%) and with IL-5 only (60%)) (*p* = 0.000001) clones in peripheral blood are significantly higher in those experiencing successful pregnancy compared to those experiencing URA (Figure 5). Moreover, as for decidua, the percentage of Th17/Th1/IL-22+ clones+ (producing IFN-γ, IL-17A, IL-17F, and IL-22, without IL-4) (*p* = 0.0001) in peripheral blood of those experiencing URA is significantly higher compared to peripheral blood of those experiencing successful pregnancy (Figure 5).

These results indicate that four subpopulations of CD4+ cells, producing IL-22, which produce IL-4 and/or IL-5 and/or IL-13, named Th0/IL-22+, Th2/IL-22+, Th17/Th0/IL-22+, and Th17/Th2/IL-22+ cells, are associated with the success of pregnancy, whereas the only subpopulation of CD4+ cells associated with URA, which produces IL-22, the Th17/Th1/IL-22+ cells, does not produce IL-4. The associated production of IL-22 and IL-4, as previously shown, seems to be essential for the success of pregnancy. IL-22 produced by decidual CD4+ T cells, if associated with IL-4 (and/or IL-13 and IL-5) production, is not deleterious for pregnancy outcome. 

### 2.6. Th17/Th2/IL-22+ and Th17/Th0/IL-22+ CD4+ T Cells Are Exclusively Present at the Implantation Site of Ectopic Pregnancy

Decidual Th0/IL-22+, Th2/IL-22+, Th17/Th0/IL-22+, and Th17/Th2/IL-22+ cells seem to be important for successful pregnancy development. We wondered whether these cells were present at the implantation site of the embryo and thus could have an important role for embryo implantation. To answer this question, we performed the cytokine analysis in ectopic tubal pregnancies.

We evaluated the percentages of Th0/IL-22+, Th2/IL-22+, Th17/Th0/IL-22+, Th17/Th2/IL-22+, and Th17/Th1/IL-22+ cells among the CD4+ T cell clones (*N* = 67) respectively derived from the implantation site of the embryo and distant from the implantation site in the same Fallopian tube of four women suffering from ectopic pregnancy (Experiment 2 according to Section 4.3). 

**Figure 6:**
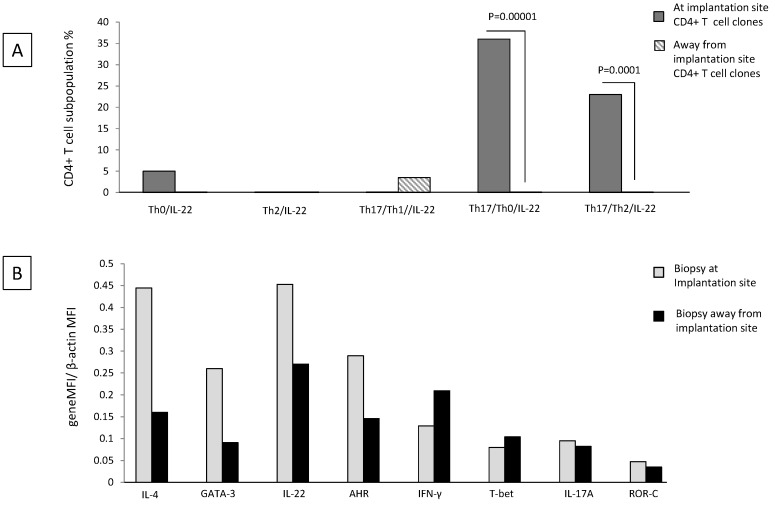
Th17/Th2/IL-22+ and Th17/Th0/IL-22+ CD4+ T cells are exclusively present at the implantation site of ectopic pregnancy. The percentages of Th0/IL-22+, Th2/IL-22+, Th17/Th0/IL-22+, Th17/Th2/IL-22+, and Th17/Th1/IL-22+ cells among the CD4+ T cell clones respectively derived from the implantation site of the embryo and distant from the implantation site in the same Fallopian tube of four women suffering from ectopic pregnancy were evaluated (Experiment 2 according to Section 4.3). The statistical analysis was performed with the chi-square test. The determination of mRNA level for IL-4, GATA-3, IL-17A, ROR-C, IL-22, AHR, T-bet, and IFN -γ in Fallopian tube tissue taken at the embryo implantation site and tissue sampled distant from the implantation site of an additional woman suffering from ectopic pregnancy was performed by Quantigene 2.0.

There is no significant difference in the percentage of Th0/IL-22+, Th2/IL-22+, and Th17/Th1/IL-22+ CD4+ T cell clones generated from the implantation site and distant from the implantation site (Figure 6A) (although the last subpopulation seems to be prevalent apart from the implantation site and the first subpopulation seems to be prevalent at the implantation site). At the implantation site, the percentage of Th17/Th0/+IL-22+ (producing IFN-γ, IL-4, IL-5, IL-13, IL-17A, IL-17F, and IL-22 (86%), producing IFN-γ, IL-4, IL-17A, IL-17F, and IL-22 (7%), producing IFN-γ, IL-4, IL-13, IL-17A, IL-17F, and IL-22 (7%)) (*p* = 0.00001) and the percentage of Th17/Th2/IL-22+ (producing IL-4, IL-17A, IL-17F, and IL-22 with IL-5 and IL-13 (67%) and with IL-13 only (33%)) (*p* = 0.0001) CD4+ T cell clones are higher than that of those clones derived distant from the implantation site (Figure 6A). 

We confirmed these results by determining the mRNA level of IL-4, GATA-3, IL-17A, ROR-C, IL-22, AHR, T-bet, and IFN−γ in Fallopian tube tissues taken at the embryo implantation site and tissue sampled distant from the implantation site of an additional woman suffering from ectopic pregnancy (Figure 6B). At the implantation site, the levels of mRNA for IL-22 and its associated transcription factor AHR, for Th2-type molecules (IL-4 and GATA3) and for Th17-type molecules (IL-17A and RORC), are increased compared to the mRNA levels for these molecules apart from the implantation site. In contrast, away from the implantation site, the mRNA production level of IFN-γ and its associated transcription factor T-bet is increased compared to those expressed at the embryo implantation site (Figure 6B). 

**Figure 7:**
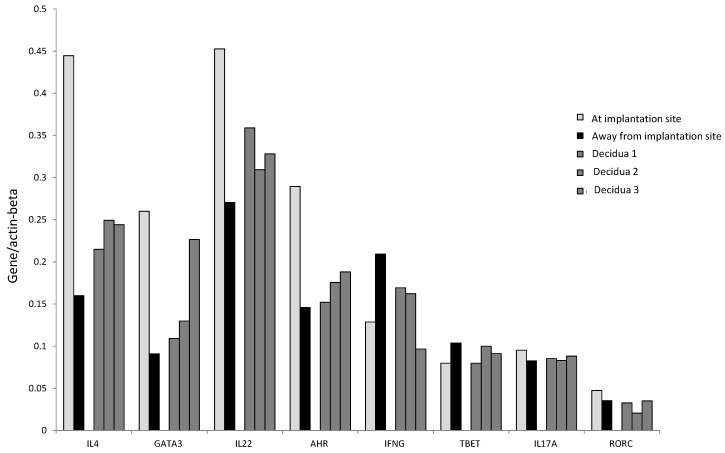
The determination of mRNA level for IL-4, GATA-3, IL-17A, ROR-C, IL-22, AHR, T-bet, and IFN -γ in Fallopian tube tissue taken at the embryo implantation site and distant from the implantation site of a woman suffering from ectopic pregnancy and in decidual biopsies from three women with successful pregnancy was performed by Quantigene 2.0.

Interestingly, IL-4, GATA-3, IL-17A, ROR-C, IL-22, and AHR mRNA levels seem to be higher at the implantation site than those found in the decidual biopsies of successful pregnancy, indicating that the production of IL-22, IL-4, and IL-17A is concentrated at the embryo implantation site (Figure 7). By contrast, the levels of IFN-γ and T-bet were higher away from the implantation site compared to those found in the decidual biopsies of successful pregnancy and at the embryo implantation site of the tubal biopsy (Figure 7). These results indicate that IFN-γ increases when implantation fails or does not occur. 

## 3. Discussion

Trophoblast expressing paternal Class I antigens thought to resemble a semiallograft, and could be rejected by maternal CD4+ T cells. IL-22, which could be produced by CD4+ Th17 and Th22 cells, has been shown to be involved in allograft rejection, by increasing IFN-γ production by Th1 and Tc1 cells, known to be responsible for acute allograft rejection, and decreasing IL-10 production by T reg and Th2 cells, known to be responsible for allograft tolerance [26,27]. For this, it has been suggested that IL-22 at the fetal maternal interface could be responsible for spontaneous abortion. Some authors confirmed this hypothesis by reporting that, in the serum of women suffering from URA, there are increased levels of IL-22 compared to the levels of IL-22 present in the serum of women with successful pregnancy [29]. However, Roomandeh et al. (2018) [29] measured IL-22 only in the serum and did not investigate IL-22 production at fetal maternal interface, where the factors of uterine microenvironment could control the cytokine profile of decidual cells producing IL-22. 

We investigated the effect on pregnancy outcome of IL-22 produced by T helper cells in the peripheral blood and, more importantly, in the decidua of those suffering from URA (during a spontaneous abortion) and of women experiencing successful pregnancy. Our results show that the percentage of CD4+ T cells producing IL-22 (Th/IL-22+) in the decidua of those experiencing successful pregnancy (62.9%) is higher than the percentage of T helper cells in the deciduae of those experiencing URA (21.6%) (*p* = 0.002), suggesting that there is a prevalence of T helper cells producing IL-22 in the decidua of those experiencing successful pregnancy compared to those experiencing URA. 

Our results, in agreement with O’Hern Perfetto et al. (2015) [28], who reported that mRNA for IL-22 increases in decidua of those experiencing successful pregnancy compared to those experiencing URA, suggest that IL-22 produced by CD4+ T cells could have a positive effect on pregnancy and that, although IL-22 produced by CD4+ T cells could be responsible for fetoallograft rejection, IL-22 could also be beneficial for pregnancy at least in some conditions. O’Hern Perfetto et al. (2015) [28] suggested by immunohistochemistry that IL-22 could be produced by decidual NK cells, but did not investigate if these cells could be the only source of IL-22 and if IL-22 could be produced by decidual CD4+ T cells at the fetomaternal interface. 

Thus, our previous results suggested that IL-22 could also be beneficial for pregnancy at least in some conditions. The results, we will discuss below, will show the conditions under which IL-22 could be beneficial for pregnancy. In fact, we found an associated production of IL-4 and IL-22 by decidual CD4+ T cells in those experiencing successful pregnancy, not found in those experiencing URA, which was confirmed at the mRNA level in the decidua of those experiencing successful pregnancy, with the associated expression of mRNA for IL-22, IL-4, and their transcriptional factors, AHR and GATA3, respectively. In agreement with these results, we found i) that IL-22 produced by decidual CD4+ T cells of those experiencing successful pregnancy is positively correlated with IL-4 produced by the same cells, whereas, in those experiencing URA, IL-22 is positively correlated with IL-17A and IL-17F, but not with IL-4, and ii) that IL-22 is positively correlated with IL-4 in the serum of those experiencing successful pregnancy.

Furthermore, we investigated to which CD4+ T helper subpopulation(s) the decidual Th/IL-22+ cells belong and if particular T helper profiles, characterized by the associated production of IL-22 and IL-4, are preferentially associated with pregnancy failure or successful pregnancy. We analyzed the percentages of Th1-, Th2-, Th0-, Th17-, Th17/Th1-, and Th17/Th2-cells, which also produce IL-22 (Th1/IL-22+, Th2/IL-22+, Th0/IL-22+, Th17/IL-22+, Th17/Th1/IL-22+, and Th17/Th2/IL-22+). We analyzed these subpopulations not only at the decidual level but also the peripheral blood. We found that four subpopulations of CD4+ cells producing IL-22 and IL-4 (Th0/IL-22+, Th2/IL-22+, Th17/Th0/IL-22+, and Th17/Th2/IL-22+) are associated with the success of pregnancy, whereas the only subpopulation of CD4+ cells producing IL-22 and associated with URA does not produce IL-4. The associated production of IL-22 and IL-4 seems to be essential for the success of pregnancy. IL-22 produced by decidual CD4+ T cells, if associated with IL-4 production, is not deleterious for pregnancy. Thus, our results suggest that the condition under which IL-22 produced by CD4+ T cells could be beneficial for pregnancy when IL-22 production is associated with IL-4 production.

Decidual Th0/IL-22+, Th2/IL-22+, Th17/Th0/IL-22+, and Th17/Th2/IL-22+ cells seem to be important for successful pregnancy development. We wondered whether these cells were present at the implantation site of the embryo and thus could play an important role in embryo implantation. To answer this question, we performed the same kind of cytokine analysis in ectopic tubal pregnancies. We evaluated the percentages of Th0/IL-22+, Th2/IL-22+, and Th0/IL-22+ of Th2/IL-22+, Th17/Th0/IL-22+, Th17/Th2/IL-22+, and Th17/Th1/IL-22+ cells among the CD4+ T cells respectively derived from the implantation site of the embryo and distant from the implantation site in the same Fallopian tube of patients suffering from ectopic pregnancy. 

We found that, at the implantation site, the percentage of Th17/Th0/+IL-22 and of Th17/Th2/IL-22+CD4+ T cells is higher than those of T cells derived distant from the implantation site. We also confirmed the associated and increased expression of mRNA for IL-4 and IL-22 and their respective transcriptional factors, GATA3 and AHR, in the tissue derived from the embryo implantation site compared to the tissue distant from the implantation site in the Fallopian tube with ectopic pregnancy.

We are not surprised by the associated production of IL-22 and a Th2-type cytokine (IL-4) by CD4+ T cells, because Plank et al. (2017) [11] reported that in vitro Th22 cells may demonstrate a plasticity toward Th2-type cells. Under Th2-promoting conditions in vitro, as well as in vivo, Th22 cells display marked plasticity toward the production of IL-13 (another Th2-type cytokine). Consistent with these results, skin-homing IL-13- and IL-22-producing Th2/22 and Tc2/22 cells were recently found to be elevated Th2-type pathologies as atopic dermatitis (AD) [15]. A Th2/Th22 inflammatory pathway has also been reported in acute canine AD skin lesions [16,17]. We demonstrated for the first time the possible association of IL-22 and another Th2-type cytokine different from IL-13, IL-4 produced by CD4+ T cells.

**Figure 8:**
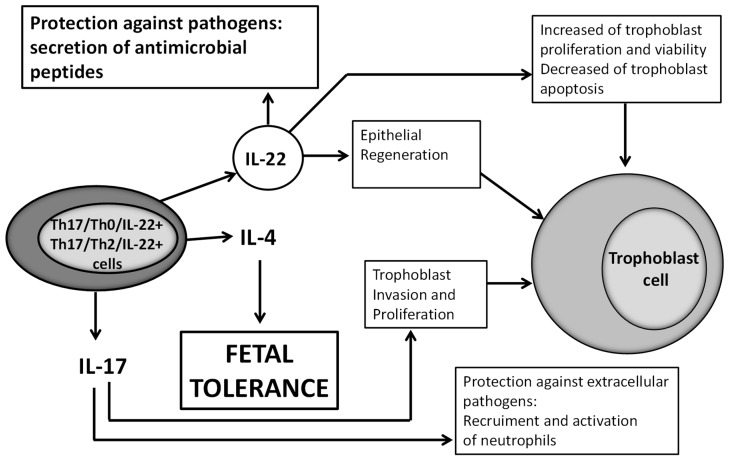
Possible positive roles of Th22/Th2/IL-22+ and Th17/Th0/IL-22+ cells at fetal maternal interface.

We could wonder what could be the beneficial role of IL-22 in pregnancy. The receptor for IL-22 (IL-22R1) is present on trophoblast cells and human villi [32]. This receptor is known to be present only on epithelial cells, and trophoblast cells are epithelial cells of fetal origin. As IL-22 is important for epithelial regeneration and wound repair [33,34], IL-22 at the fetal maternal interface could have a positive effect on pregnancy by repairing damage of the trophoblast cells. IL-22 could also be beneficial for pregnancy because it induces the proliferation and survival and decreases the apoptosis of trophoblast cells [32]. In addition, IL-22 could be beneficial for pregnancy by protecting trophoblast from pathogens, which cause a fivefold increase in the number of miscarriages in virtue of its ability to induce the secretion of antimicrobial peptides locally [35]. Very recently, in late pregnancy, in particular in preterm birth, it has been shown that IL-22 could contribute to defense against inflammatory responses at the fetal maternal interface in response to intrauterine infection [36]. In fact, the authors demonstrated that IL-22 is upregulated in uterine tissue in response to bacterial endotoxin and prevents apoptosis of placental cells. Importantly, supplementation with recombinant IL-22 significantly improved pregnancy outcomes in mice, which were challenged with intrauterine LPS (expressed by Gram-pathogens) treatment [36] (Figure 8). 

Th17/Th0/IL-22+ and Th17/Th2/IL-22+ CD4+ T cells, which are present at the embryo implantation site, produce IL-22, which could have a positive effect on pregnancy when these CD4+ T cells produce it in association with IL-4, as does IL-17 [25], which can induce trophoblast proliferation and invasion and a protection against extracellular pathogens by recruiting neutrophils. IL-22 could act positively on pregnancy by inducing epithelial regeneration [33,34] and could, at the fetal maternal interface, repair damage of the trophoblast cells. IL-22 could also be beneficial for pregnancy because it induces the proliferation and survival and decreases the apoptosis of trophoblast cells [32] and could be beneficial for pregnancy by protecting trophoblast from pathogens, responsible for miscarriages, by its ability to induce the secretion of antimicrobial peptides locally [35,36]. 

## 4. Material and Methods

### 4.1. Reagents

PHA was purchased from GIBCO Laboratories (Grand Island, NE, USA) and phorbol 12-myristate 13-acetate (PMA) from Sigma Chemical Co. (St. Louis, MO, USA). OKT3 (anti-CD3) mAb was purchased from Ortho Pharmaceuticals (Raritan, NJ, USA). Anti-CD4 and anti-CD8 were obtained from Becton-Dickinson (Mountain View, CA, USA). Human recombinant IL-2 was a generous gift from Eurocetus (Milano, Italy). FCS was from HyClone Lab Inc. (Logan, UT, USA). 

### 4.2. Subjects

Thirty pregnant women with normal gestation and no spontaneous abortion in their past history had requested elective termination were enrolled in the study with 27 women, who had histories of at least 7 ± 3 (range, 4–10) prior first-trimester spontaneous abortions, which could not be explained on the basis of conventional criteria (normal parental chromosomes, hysterosalpengography and hysteroscopy, endometrial biopsy, hormonal analysis including FSH, LH, estradiol, testosterone, cervical cultures for the presence of ureaplasma, mycoplasma and chlamydia, lupus anticoagulant, anti-phospholipid antibodies, and thyroid function tests). Specimens of deciduae and peripheral blood were obtained at the time of spontaneous abortion (at 8–11 weeks of pregnancy with normal karyotype of trophoblast), at which time all women were in excellent health, had no history of atopy or allergy, and were taking no medication. Trophoblast-invaded tubal mucosa at the implantation site and tubal mucosa distant from the implantation site were obtained from 6 women (with no history of spontaneous abortion) whose ectopic pregnancies were terminated by surgical removal as a result of threatened tubal rupture. The women agreed to participate in the study at the Hospital of Rijeka, Croatia. All subjects received verbal and written information about the aim and the design of the research, and all women provided written informed consent. The study was approved by local ethics committees of the Medical Faculty of Rijeka of the Clinical Hospital Center of Rijeka (N. 2170-29.02/1-06-1 and 2170-24-09-7-06-02). The mean age and the gestational age values of the three groups of patients (successful pregnancy, unexplained recurrent abortion, and ectopic pregnancy) were not statistically different.

### 4.3. Generation of CD4+ T-Cell Clones from Peripheral Blood, from Decidual Biopsies of Those Experiencing Successful Pregnancy and Those Experiencing URA, and from Fallopian Tube Biopsies of Ectopic Pregnancy

Specimens of deciduae (separated from villus with normal karyotype) and of Fallopian tubes were washed twice in PBS (pH 7.2) and then disrupted in small fragments (2–3 mm in diameter). Short-term T-cell lines were generated by culturing single fragments for one week in 24-well plates (Costar, Cambridge, Massachusetts) in 2 mL of RPMI 1640 supplemented with 2 mM L-glutamine, 20 M-mercaptoethanol, 10% FCS (complete medium) (Hyclone Laboratories, Logan, Utah), and IL-2 (Eurocitus, Milan, Italy) (20 U/mL). T-cell clones were then generated from short-term cultures of decidual and tubal T cells derived in the presence of IL-2, as well as from PBMC obtained from the same donors, using a method described elsewhere [31]. The phenotype of CD3+CD4+ of T-cell clones was assessed by flow cytometer analysis.

### 4.4. Induction of Cytokine Production by T-Cell Clones

To induce the cytokine production, 10^6^ T-cell blasts from each T-cell clone were cultured in the presence of PMA (20 ng/mL; Sigma, St. Louis, MO, USA) plus monoclonal antibody against CD3 (100 ng/mL; Ortho Pharmaceuticals, Raritan, New Jersey). After 36 h, culture supernatants were collected, filtered, and stored in aliquots at −80 °C.

### 4.5. Determination of Cytokine Concentrations in Supernatants with Bead-Based Multiplex Immunoassays

The quantitative determination of IL-4, IL-5, IL-13, IL-17A, and IFN-γ was performed by a bead-based multiplex immunoassay (Biorad Laboratories, Hercules, CA, USA) and the determination of IL-17F and IL-22 by another bead-based multiplex immunoassay (Millipore, Billerica, Massachusetts) using a Bioplex 200 system (Biorad Laboratories, Hercules, CA, USA), as we have previously described elsewhere [37]. In brief, supernatant was added to antibody-conjugated beads directed against the cytokines listed above in a 96-well filter plate. After a 30 min incubation, the plate was washed and biotinylated anti-cytokine antibody solution was added before another 30 min incubation. The plate was then washed and streptavidin-conjugated PE was added. After a final wash, each well was suspended with assay buffer and analyzed with the Bioplex 200 system. Standard curves were derived from various concentrations of the different cytokine standards and followed the same protocol as the supernatant samples. The concentration of each cytokine (pg/mL) in each T cell clone supernatant was calculated thanks to the Bioplex200 software. 

### 4.6. Quantification by a Multiplex Gene Assay (Quantigene 2.0) of IL-4, IL-17A, IL-17F, IL-23R, IFN-γ, RORC, GATA3, AHR, and IL-22 mRNA 

The mRNA quantization for the genes was performed with a Multiplex Gene Assay (Quantigene 2.0, Thermo Fisher, Waltham, MA, USA), as we previously described elsewhere [38,39]. Briefly, the mRNA expression of IL-4, IL-17A, IL-17F, IL-23R, IFN-γ, RORC, GATA3, AHR, IL-22, and Actb (high expression housekeeping gene) was measured using the QuantiGene multiplex assay (Thermo Fisher, Waltham, MA, USA). Samples (biopsies of decidua from those experiencing successful pregnancy and tubal biopsies of those experiencing ectopic pregnancies at the implantation site and away from the implantation site) were lysed after treatment in a lysis mixture; mRNA expression in lysates was detected and measured according to the manufacturer’s instructions. Samples were frozen in RNA later (Qiagen, Germany). Each sample was weighed, and the appropriate lysis solution was added to a final volume of 150 μL containing 50% Lysis Mixture (Thermo Fisher, Waltham, MA, USA) and 1 g/L Mixture (Thermo Fisher, Waltham, MA, USA) and 1 g/L proteinase K. The mixture was shaken at 65 °C for 30 min to lyse the cells. The lysate was stored at −80 °C for later use.

A panel of oligonucleotide capture probes, each with a unique sequence of 15 bases, was covalently linked to carboxylated fluorescently encoded beads (Luminex, Bio-rad, Massachusetts, USA). We mixed each sample lysate diluted at 1:1 and 1:2 with the pooled capture beads in a round-bottom assay well and hybridized for 16 h at 54 °C (final volume in each well was 100 μL). The assay mixture was transferred to a MultiScreen filter plate (Millipore, Billerica, MA, USA), and unbound material was filter-washed from the wells by rinsing 3 times with wash buffer. The plate was then hybridized at 54 °C for 1 h with 100 μL/well of a bDNA amplifier in Amplifier Diluent (Thermo Fisher, Waltham, MA, USA). Afterward, the plate was filter-washed twice with a wash buffer and incubated at 50 °C for 1 h with 100 μL/well of 5′-dT (Biotin)-conjugated label probe (Thermo Fisher, Waltham, MA, USA) diluted in Label Probe Diluent (Thermo Fisher, Waltham, MA, USA). After 2 washes, streptavidin-conjugated R-phycoerythrin diluted in SA-PE diluent (20 mmol/L Tris-HCl, 400 mmol/L lithium chloride, 1 mL/L Tween 20, 1 mL/L bovine serum albumin, and 5 mL/L Micr-O-protect) was added, and the plate was shaken and incubated at room temperature for 30 min. We washed the beads to remove unbound SA-PE and then analyzed them with Bio-Plex 200 system (Bio-Rad). The SA-PE fluorescence measured from each bead was proportional to the number of mRNA transcripts captured by the beads. Expression of target-specific RNA molecules was calculated as mean values from triplicate cultures and normalized against the Actin gene (high expression housekeeping gene).

### 4.7. Serum from Peripheral Blood of Those Experiencing Successful Pregnancy and URA

We analyzed the serum of 18 first-trimester women experiencing successful pregnancy who had requested elective termination and the serum of 18 women with URA, who underwent a spontaneous abortion. In all sera, isolated from 5 mL of peripheral blood and then frozen at −80 °C, the quantitative determination of IL-4, IL-13, IL-17A, IFN-γ, and IL-22 by bead-based multiplex immunoassays as we have previously described above.

### 4.8. Statistics

Statistical analyses were performed using SSPS software (SPSS, Inc, Evanston, IL, USA). Due to non-parametric distribution, all comparisons between cytokine concentrations in controls and those experiencing URA were performed by the Wilcoxon test. The subpopulations percentages were analyzed by the chi-square test. A *p*-value <0.05 was considered statistically significant.

## 5. Conclusions

As IL-17, IL-22 when produced by CD4+ T cells in association with IL-4 could be another cytokine essential for the maintenance of pregnancy. IL-22 could be essential at certain stages of pregnancy or deleterious at other stages. The chronology of action of IL-22 should be further investigated

## Figures and Tables

**Table 1:** CD4+ T subpopulations producing IL-22 in the decidua of those experiencing successful pregnancy and those experiencing URA. The percentage of the CD4+ T cell clones that produce IL-22 in the decidua of those experiencing successful pregnancy and those experiencing unexplained recurrent abortion (URA), which also produce Th2-type (IL-4, IL-5, IL-13), and/or Th1-type (IFN-γ) and/or Th17-type (IL-17A) cytokines was determined. All the cytokines were measured by a multiplex bead-based assay in the supernatant of the CD4+ T cell clones.

T Cell Clones from Decidua of URA	%	T cell Clones from Decidua of Normal Pregnancy	%	*P*
Th0/IL-22+	0	Th0/IL-22+	26	0.0002
IFN-γ+/IL-22+ IL-4+	0	IFN-γ+/IL-22+ IL-4+	0	
IFN-γ+/IL-22+ IL-4+ IL-13+ IL-5+	0	IFN-γ+/IL-22+ IL-4+ IL-13+ IL-5+	84	
IFN-γ+/IL-22+ IL-4+ IL-5+	0	IFN-γ+/IL-22+ IL-4+ IL-5+	8	
IFN-γ+/IL-22+ IL-4+ IL-13+	0	IFN-γ+/IL-22+ IL-4+ IL-13+	8	
Th2/IL-22+	0	Th2/IL-22+	17	0.0001
IL-22+ IL-4+	0	IL-22+ IL-4+	0	
IL-22+ IL-4+ IL-13+ IL-5+	0	IL-22+ IL-4+ IL-13+ IL-5+	100	
IL-22+ IL-4+ IL-5+	0	IL-22+ IL-4+ IL-5+	0	
IL-22+ IL-4+ IL-13+	0	IL-22+ IL-4+ IL-13+	0	
Th17/Th2/IL-22+	0	Th17/Th2/IL-22+	6.5	0.0005
IL17+/IL-22+ IL-4+	0	IL17+/IL-22+ IL-4+	0	
IL17+/IL-22+ IL-4+ IL-13+ IL-5+	0	IL17+/IL-22+ IL-4+ IL-13+ IL-5+	100	
IL17+/IL-22+ IL-4+ IL-5+	0	IL17+/IL-22+ IL-4+ IL-5+	0	
IL17+/IL-22+ IL-4+ IL-13+	0	IL17+/IL-22+ IL-4+ IL-13+	0	
Th17/Th0/IL-22+	2	Th17/Th0/IL-22+	13	0.04
IL-17+/IFN-γ+/IL-22+ IL-4+	0	IL-17+/IFN-γ+/IL-22+ IL-4+	0	
IL-17+/IFN-γ+/IL-22+ IL-4+ IL-13+ IL-5+	100	IL-17+/IFN-γ+/IL-22+ IL-4+ IL-13+ IL-5+	66	
IL-17+/IFN-γ+/IL-22+ IL-4+ IL-5+	0	IL-17+/IFN-γ+/IL-22+ IL-4+ IL-5+	17	
IL-17+/IFN-γ+/IL-22+ IL-4+ IL-13+	0	IL-17+/IFN-γ+/IL-22+ IL-4+ IL-13+	17	
Th17/Th1/IL-22+	22	Th17/Th1/IL-22+	0	0.005
IL-17+/IFN-γ+ /IL-22+ IL-4+	0	IL-17+/IFN-γ+/IL-22+ IL-4+	0	
IL-17+/IFN-γ+/IL-22+ IL-4+ IL-13+ IL-5+	0	IL-17+/IFN-γ+//IL-22+ IL-4+ IL-13+ IL-5+	0	
IL-17+/IFN-γ+/IL-22+ IL-4+ IL-5+	0	IL-17+/IFN-γ+/IL-22+ IL-4+ IL-5+	0	
IL-17+/IFN-γ+/IL-22+ IL-4+ IL-13+	0	IL-17+/IFN-γ+/IL-22+ IL-4+ IL-13+	0	
IL-17+/IFN-γ+/IL-22+	100	IL-17+/IFN-γ+/IL-22+	0	
